# Rare presentation of fatal atraumatic splenic rupture in follicular lymphoma

**DOI:** 10.1002/jha2.246

**Published:** 2021-06-04

**Authors:** Minqi Xu, Christine Orr, David P. LeBrun, John P. Rossiter

**Affiliations:** ^1^ Pathology and Molecular Medicine Queen's University Kingston Ontario Canada

A middle aged adult with a nearly 3‐year history of stage III follicular lymphoma experienced progressive abdominal pain and distention before collapsing suddenly. The patient was resuscitated and underwent emergency laparotomy that showed copious intraperitoneal blood originating from a rupture of a markedly enlarged spleen. Despite splenectomy, hemostasis could not be achieved and the patient was pronounced deceased.

The resected spleen was diffusely enlarged (1956 g, 27 × 18 × 9 cm) and disrupted by a large irregular laceration (Figure 1, left). Consented autopsy showed extensive mediastinal and abdominal lymphadenopathy (Figure 1, middle). Histology revealed involvement of the spleen (Figure 1, upper right and lower right), bone marrow, mediastinal, and abdominal lymph nodes by follicular lymphoma, WHO grade 1/2 out of 3. There was no evidence of transformation of the follicular lymphoma to an aggressive lymphoma.

**FIGURE 1:**
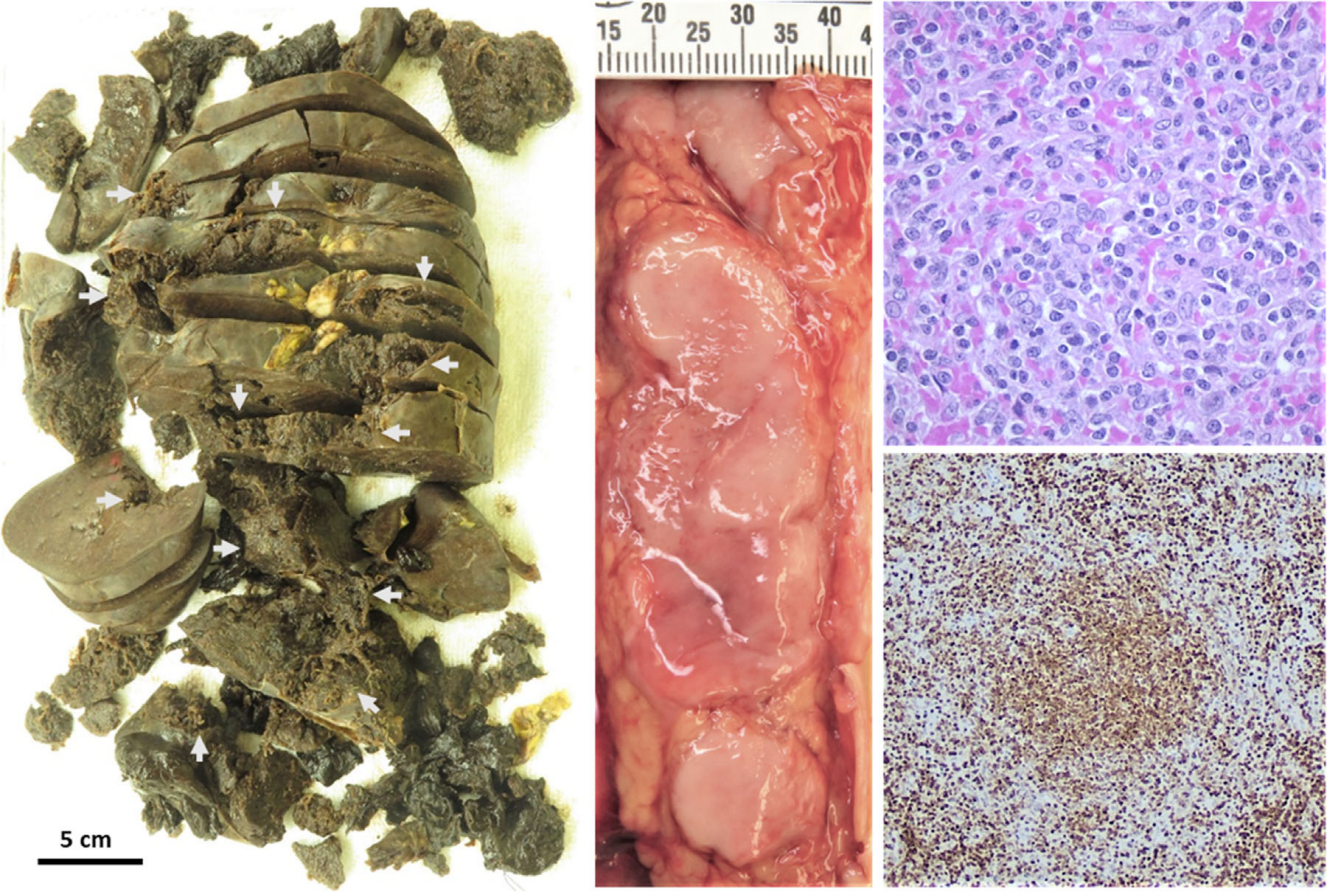
Arrows indicate lacerated areas of the diffusely enlarged spleen, photo taken after serial sectioning (left). Paraaortic lymphadenopathy (middle). Spleen involvement by low‐grade follicular lymphoma, hematoxylin phloxine saffron stain, 40× objective (upper right). One involved splenic lymphoid follicle highlighted by BCL‐2 immunohistochemistry, 10x objective (lower right)

Low‐grade follicular lymphoma is typically clinically indolent and minimally symptomatic. Atraumatic splenic rupture secondary to massive splenomegaly in follicular lymphoma is uncommon but can be fatal even with urgent medical intervention. Close monitoring for ongoing or rapid splenic enlargement and appropriate patient education may therefore be warranted in patients with follicular lymphoma.

## AUTHOR CONTRIBUTIONS

MX wrote the manuscript. CO, DL, and JR edited and revised the manuscript. All authors performed the pathologic assessment on the case reported.

## CONFLICT OF INTEREST

The authors declare no conflict of interest.

